# Sexual violence against women remains problematic and highly prevalent around the world

**DOI:** 10.1186/s12905-023-02338-8

**Published:** 2023-04-26

**Authors:** Liqing Li, Xin Shen, Guohua Zeng, Hongwei Huang, Zhensheng Chen, Jiayi Yang, Xiaofang Wang, Ming Jiang, Sule Yang, Qi Zhang, Honglang Li

**Affiliations:** 1 School of Economics and Management, Jiangxi Science and Technology Normal University, Nanchang, Jiangxi China; 2 School of Public Health, Tongji Medical College, Huazhong University of Science and Technology, Wuhan, Hubei China; 3 School of Economics and Management, Jiangxi University of Science and Technology, Ganzhou, Jiangxi China; 4 Department of Health Management Medicine, The Second Affiliated Hospital of Nanchang University, Nanchang, China

**Keywords:** Sexual violence, Women, Global prevalence, Systematic review, Meta-analysis

## Abstract

**Background:**

Sexual violence is far more prevalent in most societies than is usually suspected in daily life. However, no study has systematically summarized the global prevalence rate and the major outcomes of sexual violence against women.

**Methods:**

We directed a wide-raging search in the PubMed, Embase, and Web of Science, catalogs since the beginning to December 2022 for relevant reports about the incidence of sexual fighting touching females. The occurrence frequency was assessed with a random-effects model. The heterogeneity was estimated with *I* ^*2*^ values. Differences by research features were assessed over subgroup evaluation and meta-regression.

**Results:**

A total of 32 cross-sectional studies were included (a total of 19,125 participants). The pooled sexual violence rate was 0.29 (95% CI = 0.25–0.34). Subgroup analyses found that there was a higher rate of sexual violence against women in 2010–2019 period (0.33, 95% CI = 0.27–0.37), developing countries (0.32, 95% CI = 0.28–0.37), and interview (0.39, 95% CI = 0.29–0.49). The analysis found that more than half of women (0.56, 95% CI = 0.37–0.75) had post-traumatic stress disorder (PTSD) after experiencing sexual violence, and only a third of women considered seeking support (0.34, 95% CI = 0.13–0.55).

**Conclusions:**

Nearly one out of every three (29%) women around the world has been a victim of sexual violence in their life. This current study investigated the status and characteristics of sexual violence against women, which could provide an important reference for police and emergency health services management.

**Supplementary Information:**

The online version contains supplementary material available at 10.1186/s12905-023-02338-8.

## Background

Women's basic rights include freedom from harmless sex, and freedom from all forms of violence, coercion, or unwanted pregnancy [1]. "Any act of gender-based violence that causes or is likely to cause physical, sexual or psychological harm or suffering to a woman" is considered sexual violence against women by the United Nations and includes threats, coercion, or deprivation of liberty [2]. Although men are also victims of sexual violence, women tend to face higher rates and more severe injuries, a phenomenon that has received widespread international attention since the twenty-first century [3].

The increasing release of sexual violence-related information has provided a clearer picture of the current state of violence against women. Some of the common and serious forms of violence that women face include sexual abuse, forced prostitution, selective abortion, and neglect of girls, with sexual violence being the most widespread form [4]. Perpetrators of violence include spouses, partners, parents, other family members, neighbors, and men in positions of power or control. Some women do not experience arbitrary sexual violence in their lifetimes, while others experience it repeatedly over the course of years or even decades. Sexual violence is often the most humiliating type of gender-based violence that women are most likely to experience.

Sexual violence is far more prevalent in most societies than is usually suspected in daily life. Tolerance of sexual violence and coercion in specific social contexts makes women see it as normal and lessens condemnation [5]. Yet the prevalence of sexual violence means that globally, millions of women are experiencing this trauma and its consequences. Numerous studies have been conducted around the world to assess the prevalence of sexual violence. Most focus on specific groups of women, such as schools, colleges, or university students, clients of specific medical services, or professional groups [6]. However, no study has systematically measured the global prevalence of sexual violence against women. This study aims to provide evidence on the status and characteristics of sexual violence against women worldwide through a systematic review and meta-analysis and provide an important reference for the management of relevant humanitarian assistance medical services, which will help to help and care for women who suffer from sexual violence.

## Methods

### Search strategy and selection criteria

The meta-analysis followed the PRISMA guidelines [7, 8]. A comprehensive search including PubMed, Embase, and Web of Science databases were conducted, The time range covered inception through December 2022 related to the study topic. Manly search contention terms concluded “sexual violence” or “sexual violence” or “sexual assault”,while the search subject terms concluded “female” or “woman” or “women” or “girls.” The analysis only considered English articles published. Moreover, the reference lists of the retrieved articles were manually scrutinized for extra relevant articles.

The comprehensive search of the studies retrieved were screened by three aspects: title, abstract and a full-text assessment. If the only screening titles and abstracts could not determine whether one of studies should be involved in the analysis, the full text would be reviewed.

Potentially relevant articles were chosen by two researchers (L.L. and X.S.) based on the primary titles or abstracts. The study applied other two other researchers (G.Z. and H. H.) to review articles and build the specific data based on the following mainly criteria: (1) only concluded observational study design (cross-sectional, case–control and cohort studies); (2) the study subject defined as the female aged 14 years or higher; (3) the conception or identification of sexual violence meets the definition of United Nations to sexual violence; (4) the specially rate of sexual violence was reported by the articles or sufficient calculated information were provided. Any type of reviews, essays, letters, and commentaries were excluded.

### Data extraction

The data were extracted and compared by two separate researchers (L.L. and X.S.).The data separately ion and results comparing were performed by two researchers A predefined and standardized data extraction were used specifically from the dataset to collect information. The extraction included the sample size, research country, author names, and publication years. The post-traumatic stress disorder (PTSD) and seeking support situdation as the related outcomes were regarded.

### Quality assessment

An eleven-item index was used to assess the quality of studies in the final dataset, which was recommended by the Agency for Healthcare Research and Quality. The sample selection methodology, variables and analytical methods were assessed by specific items respectively. The response options covered two aspect yes and no (or unsure), which assigned one point and zero points. The total scores ranged from 0 to 11 points of every study. The higher scores indicated higher quality. Supplement Table 1 reports the specific scores.

### Data analysis

The rate was calculated using the random effects model. The *I* ^*2*^ was used as estimate the extent of statistical heterogeneity. The cut-off points were values at 25 percent, 50 percent, and 75 percent, which respectively meant low, moderate, or high heterogeneity [9]. The sources of heterogeneity were estimated by sensitivity analyses. The variations of rates were tested by potential factors, including year of publication, location, and survey method. Meta-regression analyses was used in all the group differences were tested in [10].

Egger’s regression test was reported to show the publication bias. The *P* < 0.10 was cut-off value to the publication bias. STATA 12.0 was used in all statistical analyses and tests of significance cut-off value of significance was *P* < 0.05 (two-tailed).

## Results

### Study selection

**Fig. 1:**
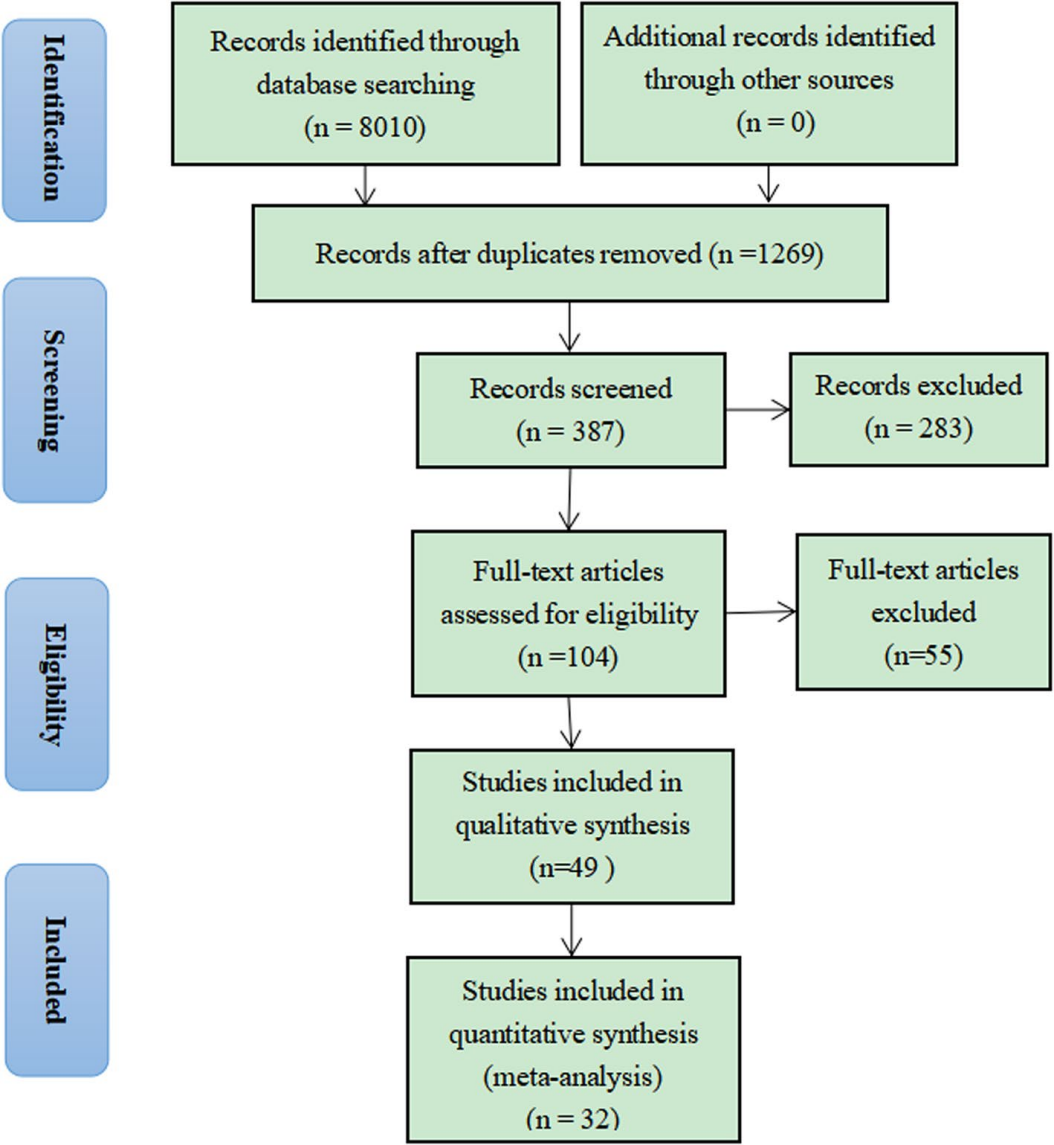
Flow chart of identification of relevant observational studies

Preferred Reporting Items for Systematic Reviews and Meta-Analyses flow chart is illustrated the study selection process in Fig. 1. In the initial screening, 8,010 articles were retrieved from PubMed, Embase and Web of Science. The detailed full-text evaluation included 104 articles remained for full-text assessment and 49 studies were left and comprised the analytical sample. Among them, 15 data insufficient articles’ data and 2 same content articles were exclued resulting in The 32 articles were included in the quantitative synthesis, which were published in 2001–2022 [11–41].

### Article and study characteristics

Table 1 showed the characteristics of these included articles. Six studies, seven studies and other seven studies were conducted in North America, Europe and Asia respectively. And three studies and nine studies were conducted in Latin America, and Africa. The sample sizes ranged from 79 to 19,125.

**Table 1:** Characteristics of studies included in the meta-analysis

**Author**	**Year**	**Country**	**Age at baseline, years**	**Survey method**	**No. of participants**
Lira et al.	2001	Mexico	≥ 14	Questionnaire	345
Jeremy et al.	2003	Britain	≥ 15	Questionnaire	1207
Bengtsson et al.	2005	Sweden	≥ 14	Questionnaire	1382
Linda et al.	2006	America	≥ 20	Interview	634
Suzanne et al.	2006	America	≥ 14	Questionnaire	898
Lilia et al.	2007	Brazil	15–49	Questionnaire	2128
Iryna et al.	2007	Uganda	15–24	Interview	3422
Shakunatala et al.	2008	India	≥ 14	Interview	2000
Nicole et al.	2009	Brazil	≥ 14	Interview	179
Schroll et al.	2010	Denmark	≥ 14	Questionnaire	2638
Elli et al.	2011	Indonesia	15–49	Questionnaire	765
Parveen et al.	2012	Cameroon	≥ 14	Questionnaire	600
Hannah et al.	2012	Ireland	≥ 18	Interview	1591
Williams et al.	2013	America	18–44	Questionnaire	9466
Matthew et al.	2014	America	≥ 14	Interview	6360
Margaret et al.	2014	Nigeria	≥ 14	Questionnaire	413
Verelst et al.	2014	Congo	11–23	Questionnaire	1305
Jill et al.	2014	America	≥ 18	Interview	432
Tanvir et al.	2014	Bangladesh	≥ 14	Interview	226
Ulla et al.	2015	Sweden	≥ 14	Questionnaire	79
Stephen et al.	2015	Uganda	≥ 15	Questionnaire	1307
Jennifer et al.	2015	Gambia	≥ 16	Questionnaire	251
Akashi et al.	2016	Rwanda	≥ 14	Questionnaire	921
Gizem et al.	2016	TurQuie	≥ 18	Questionnaire	320
Monika et al.	2016	Nepal	≥ 14	Interview	404
Ines et al.	2017	Spain	≥ 16	Questionnaire	10,171
Malin et al.	2017	India	15–49	Questionnaire	19,125
Saifa et al.	2018	Bangladesh	15–49	Questionnaire	3933
Elfalet et al.	2018	Ethiopia	18–49	Questionnaire	450
Robert et al.	2019	America	15–45	Questionnaire	292
Kiran et al.	2021	Nepal	15–24	Questionnaire	560
Bikila et al.	2022	Ethiopia	15–45	Interview	361

The median of the sample size was 921 and the interquartile range was 361–2,000. The total number of cases was 74,165. The quality scores of the studies ranged from 4 to 9 and the median score was 7.7. (Supplementary Table 1).

### Prevalence of sexual violence against women

**Fig. 2:**
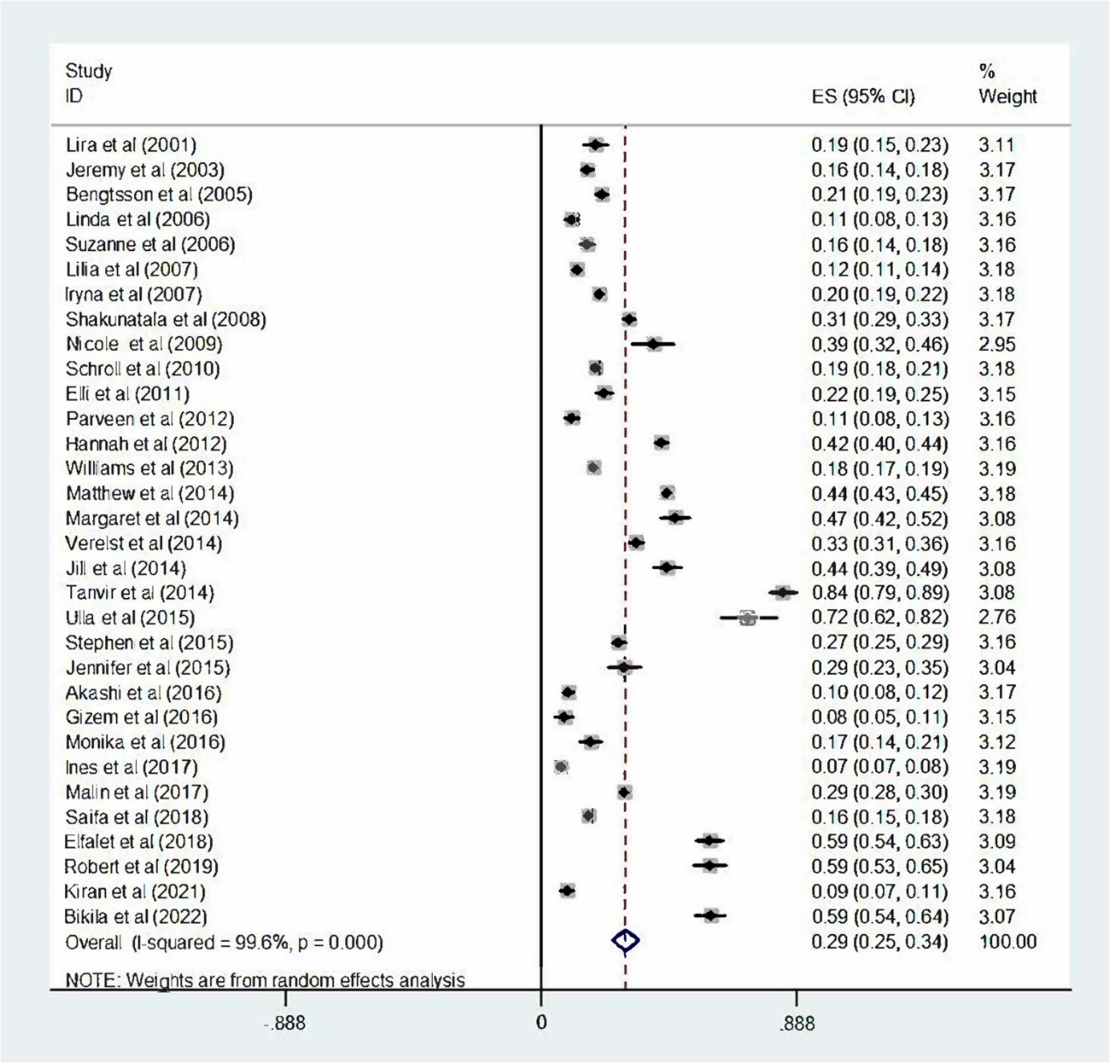
Pooled random effects prevalence rate and 95% confidence interval

The pooled rate was 0.29 (95% CI = 0.25–0.34). This result indicated that basically 29% of women had suffered from the sexual violence (*I* ^*2*^ = 99.6%, *P* < 0.0001) (Fig. 2).

### Subgroup analysis

No significant differences of study quality and study location were observed. Groups comparisons found the rate data of sexual violence obtained from 2010–2019 (0.33, 95% CI = 0.27–0.37) had a higher rate than those studies whose data obtained from 2001–2009 (0.20, 95% CI = 0.16–0.25), the rate obtained in developing country (0.32, 95% CI = 0.28–0.37) had a higher rate than the data obtained in developed country (0.27, 95% CI = 0.21–0.33), and interview (0.39, 95% CI = 0.29–0.49) had a higher rate than those whose data were obtained from the questionnaire (0.25, 95% CI = 0.20–0.29) (Table 2). Variability was studied after adding publication year, country developmental level, and survey method, and the results of the meta-regression showed that the survey method could explain 67.92% of the variability [42].

**Table 2:** Subgroups analyses of prevalence rate of sexual violence among women

	**No. of studies**	**Prevalence rate (%)**	**Lower Limit (LL)**	**Upper Limit (UL)**	***I*** ^***2***^ ** (%)**	***P*** ** for heterogeneity**	***P*** value* between groups
**Primary analysis**	32	0.29	0.25	0.34	99.60%	< 0.001	0.006
**Subgroup analyses**							
**Publication year**							
2001–2009	9	0.20	0.16	0.25	97.40%	< 0.001	0.027
2010–2022	23	0.33	0.27	0.39	99.70%	< 0.001	
**Study location**							
North America	6	0.32	0.19	0.45	99.70%	< 0.001	0.192
Europe	7	0.26	0.16	0.45	99.50%	< 0.001	
Asia	7	0.30	0.21	0.39	99.60%	< 0.001	
Latin America	3	0.23	0.11	0.35	99.60%	< 0.001	
Africa	9	0.33	0.23	0.42	99.10%	< 0.001	
**Developmental level**							
Developed country	19	0.27	0.21	0.33	99.70%	< 0.001	0.032
Developing country	13	0.32	0.28	0.37	99.30%	< 0.001	
**Survey method**							
Questionnaire	22	0.25	0.20	0.29	99.50%	< 0.001	0.011
Interview	10	0.39	0.29	0.49	99.50%	< 0.001	
**Studies quality**							
< 8	18	0.31	0.25	0.38	99.70%	< 0.001	0.053
≥ 8	14	0.27	0.20	0.33	99.40%	< 0.001	

^*^ *P* values for meta-regression

### Outcomes associated with sexual violence against women

Four articles reported the outcome associated with sexual violence against women [28, 32, 35, 36]. The analysis found that the major outcomes were post-traumatic stress disorder (PTSD) (0.56, 95% CI = 0.37–0.75), and seeking support (0.34, 95% CI = 0.13–0.55) after being exposed to sexual violence (Table 3).

**Table 3:** Meta-analysis of outcomes associated with sexual violence against women

**Outcome**	**No. of studies**	**Prevalence rate (%)**	**Lower Limit (LL)**	**Upper Limit (UL)**	***I*** ^***2***^ ** (%)**	***P*** ** for heterogeneity**
Post-traumatic stress disorder	2	0.56	0.37	0.75	89.50%	0.002
Seeking support	2	0.34	0.13	0.55	97.60%	< 0.001

### Sensitivity analyses

Sensitivity examination were made to investigate the impending causes of heterogeneity. The mutual erotic violence frequency was not substantially altered in the leave-one-out evaluates by skipping single research sequentially from 0.27 (95%CI: 0.23–0.30, *I* ^*2*^ = 91.3%) to 0.34 (95%CI: 0.32–0.36, *I* ^*2*^ = 99.7%).

### Publication bias

**Fig. 3:**
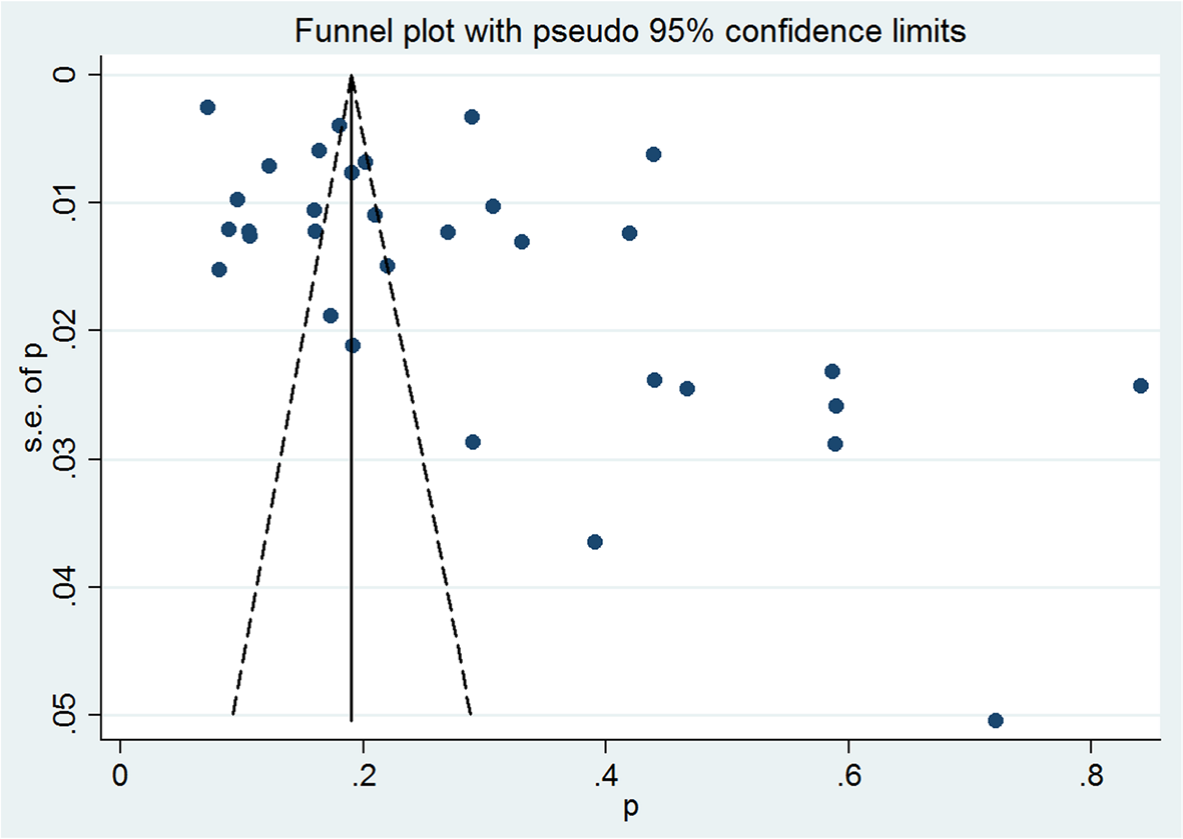
Funnel plot of the prevalence of sexual violence

A funnel plot was generated (Fig. 3) and a visual inspection of it revealed no asymmetry. Egger’s test result found no evidence of substantial publication bias (*P* = 0.25).

## Discussion

This comprehensive systematic review and meta-analysis on the global prevalence of sexual violence against women estimated that nearly one out of every three (0.29, 95% CI = 0.25–0.34) women around the world have been a victim of sexual violence in their life. The finding suggested that the high prevalence of sexual violence should be considered an important aspect of women's health care programs. Sexual violence involves serious violations of women's human rights and has adverse consequences for the physical and mental health of victims. The high incidence of this event and urgent care following it deserves more attention and is one of the key neglected needs in healthcare.

Subgroup analyses showed the rate from 2010–2019 (0.33, 95% CI = 0.27–0.37) is much higher than from 2001–2009 (0.20, 95% CI = 0.16–0.25), which indicates that sexual violence against women is increasingly becoming an important social issue. Meanwhile, developing country (0.32, 95% CI = 0.28–0.37) have a much higher sexual violence rate than developed country (0.27, 95% CI = 0.21–0.33), which indicates that the sexual violence rate may rise in countries with weaker economic fundamentals.

The group comparisons regarding the data survey method revealed a higher sexual violence rate in the studies that used interviews (0.39, 95% CI = 0.29–0.49) than those that used questionnaire data (0.25, 95% CI = 0.20–0.29) [36]. It was possible that women were more truthful in interviews than on questionnaires or that the survey questions poorly reflected the concept of sexual violence [43]. Therefore, researchers should carefully evaluate the validity of the questions in their surveys and consider multiple ways to obtain accurate data to reflect the real situation [44].

Sexual violence can produce a variety of psychological damage, and the consequences are also different. However, no woman would remain indifferent to such violations [32]. One of the most prominent reflections is a post-traumatic stress disorder. Such traumas are variable in duration and cause victims to remember past scenes, nightmares, and self-isolation, as well as increased use of drugs and alcohol, and increased frequency of risk-taking or escape activities [36]. More than half of the women (0.56, 95% CI = 0.37–0.75) developed PTSD following sexual violence, which may be the most severe psychological impact of sexual assault causing this group to be more vigilant, irritable, and emotionally explosive.

Some studies indicated that the aggressor is more frequently a member or a friend of the family, a neighbor, or another person known to the victim. However, only a fraction of all victims sought help (0.34, 95% CI = 0.13–0.55) [19]. Some studies have found that when women are sexually assaulted by someone close to them, they tend to hide it or only tell other people close to them. As a result, most incidents did not leave reports of assaults and health care records [24]. This phenomenon leads to the under-recording of such events, which may also reduce the level of attention paid to such events [27].

Sexual violence occurs in a variety of locations, including the home, school, and workplace. The perpetrators may be their husbands, relatives or strangers [25]. And the frequency is difficult to determine because there are so few events reported [24]. This type of behavior has serious implications for women's health [45]. Psychological consequences include PTSD, anxiety, and suicidal thoughts. Physical consequences include genital trauma, unintended pregnancy, and various sexually transmitted infections [38]. Therefore, prevention and care of sexual violence must be taken seriously. Initiatives should be multidisciplinary and include psychological support, treatment of physical impairment, emergency contraception, and prevention of sexually transmitted infections [41].

### Strengths and limitations

This lack of reporting was one of the reasons why sexual violence remains invisible to many health care providers who did not realize how prevalent it is. This study is the first one to investigate the global prevalence rate and mainly outcomes associated with sexual violence against women. It offers a broad perspective on the health care providers worldwide and reminds us that we should be more alert to the signs of sexual violence against women.

Though, this report has some confines to reflect when taking the findings. First, there is huge variability in the occurrence found in separate studies. This difference may be recognized as the sort of residents comprised in the analysis, socioeconomic variety, and the classifications of aggression. Many females, for example, may not state wedded assault, receiving communal standards that enforce on a partner the obligation to meet the sexual requests of their spouse. Second, great heterogeneity was detected in this study when the evaluations were combined. This heterogeneity may link to alterations in assessment approaches, sample scope, and social circumstances; nevertheless, the heterogeneity can be overvalued when reports with sizable model sizes are combined [46].

## Conclusions

Nearly one out of every three (29%) women around the world have been a victim of some type of sexual violence in their life. In summary, the results of this meta-analysis indicate that the sexual violence against worldwide women is moderate. Prevention strategies should be developed urgently to protect women from aggression.

## Supplementary Information

**Additional file 1: Supplementary Table 1.** Quality assessment of cross-sectional studies*.

## Data Availability

The datasets used and/or analysed during the current study available from the corresponding author on reasonable request.

## Abbreviation

PTSD: Post-traumatic Stress Disorder
