# Supplementary material for: Sexual violence against women remains problematic and highly prevalent around the world
Source: BMC Womens Health. 2023 Apr 26;23:196. doi: 10.1186/s12905-023-02338-8 (PMC10134525; doi:10.1186/s12905-023-02338-8)
Supplement: Supplementary file 1 — Additional file 1: Supplementary Table 1. Quality assessment of cross-sectional studies*. [file 12905_2023_2338_MOESM1_ESM.docx]

**Supplementary Table 1.Quality assessment of cross-sectional studies***

| **Author** | **Year** | **1) Define the source of information (survey, record review)** | **2) List inclusion and exclusion criteria for exposed and unexposed subjects (cases and controls) or refer to previous publications** | **3) Indicate time period used for identifying patients** | **4) Indicate whether or not subjects were consecutive if not population-based** | **5) Indicate if evaluators of subjective components of study were masked to other aspects of the status of the participants** | **6) Describe any assessments undertaken for quality assurance purposes** | **7) Explain any patient exclusions from analysis** | **8) Describe how confounding was assessed and/or controlled.** | **9) If applicable, explain how missing data were handled in the analysis** | **10) Summarize patient response rates and completeness of data collection** | **11) Clarify what follow-up, if any, was expected and the percentage of patients for which incomplete data or follow-up was obtained** | **Total quality score** |
| --- | --- | --- | --- | --- | --- | --- | --- | --- | --- | --- | --- | --- | --- |
| Lira et al | 2001 | 1 | 0 | 1 | 0 | 1 | 0 | 0 | 0 | 0 | 1 | 0 | 4 |
| Jeremy et al | 2003 | 1 | 0 | 1 | 0 | 1 | 1 | 0 | 1 | 1 | 1 | 0 | 7 |
| Bengtsson et al | 2005 | 1 | 0 | 1 | 0 | 1 | 0 | 0 | 0 | 1 | 1 | 0 | 5 |
| Linda et al | 2006 | 1 | 0 | 1 | 0 | 1 | 1 | 1 | 0 | 1 | 1 | 1 | 8 |
| Suzanne et al | 2006 | 1 | 0 | 1 | 1 | 1 | 1 | 0 | 1 | 0 | 1 | 1 | 8 |
| Lilia et al | 2007 | 1 | 1 | 1 | 0 | 1 | 1 | 0 | 1 | 1 | 1 | 0 | 8 |
| Iryna et al | 2007 | 1 | 1 | 1 | 1 | 1 | 0 | 1 | 1 | 0 | 1 | 0 | 8 |
| Shakunatala et al | 2008 | 1 | 1 | 1 | 0 | 1 | 1 | 0 | 0 | 1 | 1 | 1 | 8 |
| Nicole et al | 2009 | 1 | 1 | 1 | 0 | 1 | 1 | 0 | 1 | 1 | 1 | 1 | 9 |
| Schroll et al | 2010 | 1 | 0 | 1 | 0 | 1 | 1 | 0 | 1 | 0 | 1 | 0 | 6 |
| Elli et al | 2011 | 1 | 1 | 1 | 0 | 1 | 1 | 0 | 1 | 1 | 1 | 0 | 8 |
| Parveen et al | 2012 | 1 | 1 | 1 | 1 | 1 | 0 | 0 | 1 | 0 | 1 | 1 | 8 |
| Hannah et al | 2012 | 1 | 0 | 1 | 0 | 1 | 0 | 0 | 0 | 1 | 1 | 1 | 6 |
| Williams et al | 2013 | 1 | 1 | 1 | 0 | 1 | 1 | 1 | 0 | 1 | 1 | 1 | 9 |
| Matthew et al | 2014 | 1 | 1 | 1 | 0 | 1 | 1 | 0 | 0 | 1 | 1 | 1 | 8 |
| Margaret et al | 2014 | 1 | 1 | 1 | 0 | 1 | 0 | 1 | 1 | 1 | 1 | 1 | 9 |
| Verelst et al | 2014 | 1 | 0 | 1 | 1 | 1 | 1 | 0 | 1 | 1 | 1 | 1 | 9 |
| Jill et al | 2014 | 1 | 1 | 1 | 0 | 1 | 1 | 0 | 1 | 1 | 1 | 1 | 9 |
| Tanvir et al | 2014 | 1 | 0 | 1 | 0 | 1 | 0 | 0 | 1 | 0 | 1 | 0 | 5 |
| Ulla et al | 2015 | 1 | 0 | 1 | 0 | 1 | 1 | 0 | 1 | 0 | 1 | 1 | 7 |
| Stephen et al | 2015 | 1 | 1 | 1 | 0 | 1 | 0 | 0 | 0 | 1 | 1 | 1 | 7 |
| Jennifer et al | 2015 | 1 | 1 | 1 | 0 | 1 | 1 | 1 | 1 | 0 | 1 | 0 | 8 |
| Akashi et al | 2016 | 1 | 0 | 1 | 1 | 1 | 0 | 0 | 0 | 0 | 1 | 1 | 6 |
| Gizem et al | 2016 | 1 | 1 | 1 | 0 | 1 | 0 | 1 | 0 | 1 | 1 | 1 | 8 |
| Monika et al | 2016 | 1 | 1 | 1 | 0 | 1 | 1 | 1 | 1 | 0 | 1 | 1 | 9 |
| Ines et al | 2017 | 1 | 1 | 1 | 1 | 1 | 0 | 0 | 1 | 1 | 1 | 1 | 9 |
| Malin et al | 2017 | 1 | 1 | 1 | 0 | 1 | 1 | 0 | 1 | 1 | 1 | 0 | 8 |
| Saifa et al | 2018 | 1 | 1 | 1 | 0 | 1 | 0 | 1 | 1 | 0 | 1 | 1 | 8 |
| Elfalet et al | 2018 | 1 | 1 | 1 | 1 | 1 | 0 | 0 | 1 | 1 | 1 | 1 | 9 |
| Robert et al | 2019 | 1 | 1 | 1 | 1 | 1 | 1 | 0 | 1 | 0 | 1 | 0 | 8 |
| Kiran et al | 2021 | 1 | 1 | 1 | 0 | 1 | 0 | 1 | 1 | 1 | 1 | 1 | 9 |
| Bikila et al | 2022 | 1 | 1 | 1 | 0 | 1 | 1 | 0 | 1 | 1 | 1 | 1 | 9 |
| *The study quality was assessed according to the 11 items recommended by the Agency for Healthcare Research and Quality (AHRQ) for cross-sectional studies. 1 point if the item was contemplated in the study, 0 point if the item was not, and unable to determine. 1 = “Yes”, 0 = “No”, “Unable to determine”, or “Not applicable” | | | | | | | | | | | | | |
